# Apelin-13 administration allows for norepinephrine sparing in a rat model of cecal ligation and puncture-induced septic shock

**DOI:** 10.1186/s40635-024-00650-7

**Published:** 2024-08-05

**Authors:** William Salvail, Dany Salvail, Frédéric Chagnon, Olivier Lesur

**Affiliations:** 1 Centre de Recherche Clinique du CHU Sherbrooke (CRCHUS), CHUS, Faculté de Médecine et des Sciences de la Santé, Université de Sherbrooke, Sherbrooke, QC Canada; 2 Département de Soins Intensifs et Service de PneumologieCHUS, Faculté de Médecine et des Sciences de la Santé, Université de Sherbrooke, 3001 12th Avenue Nord, SherbrookeSherbrooke, QC J1H 5N4 Canada; 3 Département de Médecine, CHUS, Faculté de Médecine et des Sciences de la Santé, Université de Sherbrooke, Sherbrooke, QC Canada; 4 IPS Therapeutique Inc., Sherbrooke, QC Canada

**Keywords:** Apelin, Norepinephrine, Septic shock, Experimental model, Cecal ligation and puncture

## Abstract

**Background:**

Infusion of exogenous catecholamines (i.e., norepinephrine [NE] and dobutamine) is a recommended treatment for septic shock with myocardial dysfunction. However, sustained catecholamine infusion is linked to cardiac toxicity and impaired responsiveness. Several pre-clinical and clinical studies have investigated the use of alternative vasopressors in the treatment of septic shock, with limited benefits and generally no effect on mortality. Apelin-13 (APL-13) is an endogenous positive inotrope and vasoactive peptide and has been demonstrated cardioprotective with vasomodulator and sparing life effects in animal models of septic shock. A primary objective of this study was to evaluate the NE-sparing effect of APL-13 infusion in an experimental sepsis-induced hypotension.

**Methods:**

For this goal, sepsis was induced by cecal ligation and puncture (CLP) in male rats and the arterial blood pressure (BP) monitored continuously via a carotid catheter. Monitoring, fluid resuscitation and experimental treatments were performed on conscious animals. Based on pilot assays, normal saline fluid resuscitation (2.5 mL/Kg/h) was initiated 3 h post-CLP and maintained up to the endpoint. Thus, titrated doses of NE, with or without fixed-doses of APL-13 or the apelin receptor antagonist F13A co-infusion were started when 20% decrease of systolic BP (SBP) from baseline was achieved, to restore SBP values ≥ 115 ± 1.5 mmHg (baseline average ± SEM).

**Results:**

A reduction in mean NE dose was observed with APL-13 but not F13A co-infusion at pre-determined treatment time of 4.5 ± 0.5 h (17.37 ± 1.74 µg/Kg/h [APL-13] vs. 25.64 ± 2.61 µg/Kg/h [Control NE] vs. 28.60 ± 4.79 µg/Kg/min [F13A], P = 0.0491). A 60% decrease in NE infusion rate over time was observed with APL-13 co-infusion, (*p* = 0.008 vs NE alone), while F13A co-infusion increased the NE infusion rate over time by 218% (*p* = 0.003 vs NE + APL-13). Associated improvements in cardiac function are likely mediated by (i) enhanced left ventricular end-diastolic volume (0.18 ± 0.02 mL [Control NE] vs. 0.30 ± 0.03 mL [APL-13], *P* = 0.0051), stroke volume (0.11 ± 0.01 mL [Control NE] vs. 0.21 ± 0.01 mL [APL-13], *P* < 0.001) and cardiac output (67.57 ± 8.63 mL/min [Control NE] vs. 112.20 ± 8.53 mL/min [APL-13], *P* = 0.0036), and (ii) a reduced effective arterial elastance (920.6 ± 81.4 mmHg/mL/min [Control NE] vs. 497.633.44 mmHg/mL/min. [APL-13], *P* = 0.0002). APL-13 administration was also associated with a decrease in lactate levels compared to animals only receiving NE (7.08 ± 0.40 [Control NE] vs. 4.78 ± 0.60 [APL-13], *P* < 0.01).

**Conclusion:**

APL-13 exhibits NE-sparing benefits in the treatment of sepsis-induced shock, potentially reducing deleterious effects of prolonged exogenous catecholamine administration.

## Introduction

Sepsis is a life-threatening clinical condition responsible for almost 20% of worldwide deaths in 2017 [1]. Severe sepsis can rapidly lead to an even more severe clinical state with shock, exemplified by severe hypotension, elevated lactate levels and vasopressor therapy requirements [1]. With a mortality rate up to 70% in the most severe cases [2], septic shock is a burden for intensive care units (ICU), characterized by excessive immune activation and reactive oxygen and nitrogen species production compromise vascular integrity, leading to systemic vascular resistance drop, hypotension, as well as capillary hyperpermeability with tissue edema [3–5]. Together with the circulatory system, the heart is one the most prevalent dysfunctional organ during early sepsis time-course. Sepsis-induced myocardial dysfunction (SIMD) is reported in about 60% of septic shock patients and can be characterized by systolic and diastolic dysfunctions of both left and right ventricles (LV, RV) [6]. Numerous and complex bio-pathological events are involved in SIMD which is, however, potentially reversible [7–9]. A centrality of SMID and hypotension during septic shock has made cardiovascular dysfunction therapy one of the key elements of sepsis management.

Indeed, restoring adequate circulating blood volume and pressure is a cornerstone in the bundle of unavoidable interventions for septic shock improvement [10]. However, studies have shown that fluid therapy was unsuccessful in correcting hypotension in 50% of cases, justifying the use of vasopressor therapy [10, 11]. Norepinephrine (NE) is the recommended vasopressor drug administered to septic shock patients with persistent hypotension despite appropriate fluid therapy, and sometimes administered in concomitance with dobutamine (DOB), which aims at additionally improving cardiac function in SIMD patients [10]. Unfortunately, catecholamines have also shown limited therapeutic potential in the context of septic shock. Not only is responsiveness observed in only 1/3 of patients, but successive clinical trials have also shown that higher doses of DOB were not associated with lower, but rather higher mortality in septic patients [12–15]. Combined to findings regarding the deleterious effects of catecholamines on mitochondrial and cardiomyocyte function, and the oxidative stress generated through their spontaneous oxidation, these studies clearly indicate that NE and DOB do not represent ideal drugs in the context of septic shock [16, 17].

Encompassing one recognized receptor, the apelin -APJ- receptor, and several ligands, the apelin system is candidate to represent an alternative to DOB. Thanks to a wide distribution in tissues, apelins or apelin isoforms (APLs), and more recently Elabela/Toddler (Ela), have been shown to mediate myriad of physiological effects, but their real appeal remains the effects on the cardiovascular system [18, 19]. Several in vivo animal studies have reported a positive inotropic effect of APLs, along with increased ventricular elastance, left ventricular developed pressure (LVdP) and cardiac output (CO)  [20, 21]. Importantly, those effects were neither associated to an increased heart rate (HR), nor to hypertrophy of ventricles [20, 21]. We have already shown that septic rats infused with APL-13 and Ela resulted have improved survival, and that the cardiovascular effects of APL-13 were superior and more sensitive than those of DOB in the context of experimental septic shock [22, 23].

Considering the potential of APLs as novel septic shock therapy, this study aimed at evaluating the NE-sparing effect of APL-13, the most dominant and shortest active isoform detected in human heart, vessels, and bloodstream, in a context of experimental septic shock. We hypothesized that co-administration of APL-13 and NE to animals in a state of septic shock would lead to a decrease in cumulative NE doses when compared to animals not receiving APL-13, by means of an improved cardiac function [21, 24].

## Materials and methods

### Animal model of cecal ligature and puncture (CLP)-induced septic shock

**Fig. 1:**
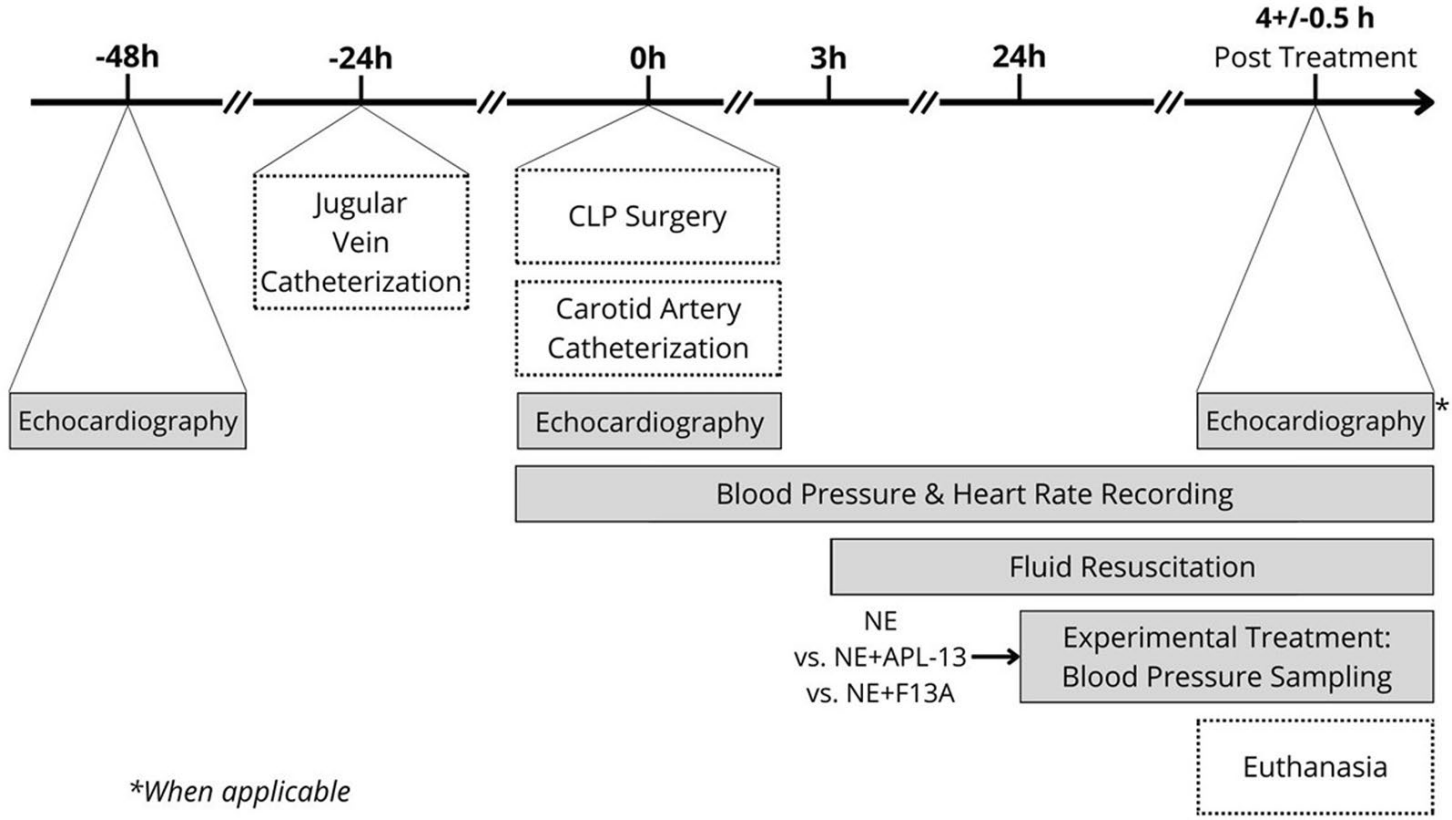
Induction of septic shock by cecal ligature and puncture (CLP) was preceded by the catheterization of the right jugular vein and followed by the catheterization of the left carotid artery. Blood pressure and heart rate were monitored throughout the experiment. Fluids were administered intravenously by continuous infusion starting 3 h post-CLP, and experimental treatments intravenously, by continuous infusion, starting 24 h post-CLP

Sprague–Dawley adult rats (300 ± 50 g, Charles River, Montreal, Can) received care in compliance with both Canadian Council of Animal Care and National Institutes of Health guidelines, and per the Animal Research: Reporting of In Vivo Experiments (ARRIVE) guidelines. All animals were housed in a temperature-controlled housing, at 22 ± 1 °C, for the entire duration of the experiments. Approval was obtained from our Institutional Ethics Review Board (#2020-2811). The study design is displayed in Fig. 1. All animals were allowed an acclimatation period of 5 days before being manipulated. Prior to any surgical procedures, baseline echocardiography measurements were obtained, and animals were transferred to a housing distinct from their initial space. They were also equipped with the harness portion of a harness-and-swivel system and allowed to acclimate to their new environment for 24 h. Septic shock was induced through the cecal ligature and puncture (CLP) procedure. A polyethylene (PE)-50 catheter was introduced in the right jugular vein 24 h prior to septic shock induction and connected to a harness-and-swivel system (Instech Laboratories, Inc., PA, USA). Each surgical procedure was preceded by a subcutaneous lidocaine injection, 10 min prior to incision, at a dose of 2 mg/Kg. The animals were transferred to clean cages following the CLP procedure, and monitored as they regained consciousness. 3 h after the CLP surgery, the animals were administered a single subcutaneous dose of slow-release buprenorphine (0.5 mg/Kg). No antibiotics were given in this protocol. To mimic fluid resuscitation priorities of clinical septic shock therapy recently upgraded [10], sterile normal saline (NS) solution was administered at a rate of 2.5 mL/Kg/min starting 3 h post-CLP, and amended to this small animal preclinical condition, as previously reported [22]. According to preliminary pilots, rats exhibiting a ≥ 20% decrease in systolic blood pressure (SBP) 20–24 h post-CLP qualified for a state of septic shock. Animals presenting a significant decrease in SBP earlier post-CLP (i.e., ≥ 20%, ≤ 20 h) as well as those not presenting a significant decrease in SBP > 24 h after CLP, were excluded from the study and euthanized. Monitoring of internal temperature using Star Oddi Temperature Data Loggers, implanted in the abdominal cavity, showed no significant difference between rats challenged or not challenged with CLP. Before-CLP data were compiled in the “Baseline” group. Qualified animals were assigned to one of four groups: Control Saline, Control NE, APL-13 or F13A in an unblinded fashion. The study was not randomized, and the attribution made by convenience, because there were no distinctive parameters allowing the response of treatment to be predicted upon inclusion. Control Saline animals only received the regular fluid resuscitation but no drugs, hence were terminated 24 h after CLP, following echocardiography measurements. Animals assigned to the other experimental groups were treated with either NE alone or in concomitance with fixed-doses of APL-13 (0.25 µg/Kg/min.) or Apelin F13A—subsequently called F13A—([Ala]-Apelin-13, 0.5 µg/Kg/min.), for a period of 4.5 ± 0.5 h. F13A is a specific apelin receptor -APJ-antagonist, with lower binding affinity, and devoid of any inotropic effect [23]. Selecting a window of monitoring time with treatment, as opposed to a fixed duration, allowed a little flexibility while respecting the endpoints imposed by the ethics protocols associated with this project. This window of time was factually a period of 4.5 ± 0.5 h. NE doses were real-time titrated to restore and sustain SBP to baseline values, and fluid infusion was maintained a constant infusion rate of 2.5 mL/Kg/h throughout the experiment. At the end of the monitoring period, the animals were anesthetized and prepared for echocardiography measurements before being euthanized.

### In vivo hemodynamic recording and measurements

Blood pressures (BP) and HR were monitored continuously in conscious animals for the duration of the experiment, through a PE-50 catheter inserted in the left carotid artery immediately following the CLP procedure. Cardiac function was evaluated through transaortic echocardiography, in this context with lightly anesthetized animals (1.2–2.0% isoflurane; 98.5–98.0% Oxygen), using General Electric’s Vivid 7/Vivid 9 equipped with the M12L ultrasonic probe (General Electric, MA, USA). Values of LV end-diastolic and LV end-systolic volumes (LVEDV, LVESV) were recorded, from which were calculated stroke volume (SV) and cardiac output (CO, mL/min). LV end-systolic pressure (ESP) was estimated using carotid pulse tracings as the ratio of dicrotic notch (*b*) to the peak (*a*): [(*b*/*a*) x pulse pressure (PP)] + diastolic BP (DBP). Also, rate pressure product (RPP = HR x SBP × 10^3^) indicative of cardiac oxygen consumption, and arterial elastance (Ea = ESP/SV) representative of the arterial load, were calculated as previously described [22, 23].

### Calculation of norepinephrine doses

Similarly to clinical practice, NE doses were titrated based on each animal’s response to the drug and associated variations in SBP. Cumulative doses of NE were calculated by multiplying the infusion rate by the infusion time, then by the NE solution concentration. To normalize for animal weight, this value was divided by the animal’ weight as per the formula below:

$$\text{Cumulative NE Dose}=\frac{\left(\left({\text{Rate}}_{1} X {\text{Time}}_{1}\right)+\left({\text{Rate}}_{2} X {\text{Time}}_{2}\right)+\left(\dots \right)\right)* \left[\text{NE} \text{Solution}\right]}{\text{BW}}$$

where Rate: infusion rate (µL/min.), Duration: duration of the infusion at the indicated rate (min.), BW: body weight (Kg).

The average NE dose was calculated by dividing the cumulative NE dose by the corresponding treatment duration, obtaining a dose expressed in µg/Kg/min.

### Statistical analysis

Results are expressed as mean ± standard error of the mean (SEM) for normally distributed datasets. *p* values < 0.05 were considered as significant. Comparisons between normally distributed groups with equal standard deviation (SD) were first analyzed by one-way analysis of variance (ANOVA) and then a Tukey’s multiple comparison test (when *p < 0.05*) for more than two group’s comparisons, or through a student *t*-test for group-by-group comparisons. Normally distributed datasets with unequal standard deviation were analyzed through Brown-Forsythe and Welch’s ANOVA test, followed by Dunnett’s T3 multiple comparisons test. Comparison of groups presenting a non-Gaussian distribution were analyzed through Kruskal–Wallis’ test, and subsequent Dunn’s multiple comparisons test (when *p* < 0.05). All figures display the results of group-by-group *t*-test comparisons. A linear mixed model was used to evaluate the differences in time and treatment between the experimental groups. Using mixed modelling is optimal when multiple timepoints are collected. As outcomes were only collected at one specific timepoint, the mixed model analysis was not applicable or useful, and simple statistical decisional tests were used. However, the analysis for infusion rate was corrected for using a linear mixed model. The dependent variables were log-transformed, and treatment arm and time were included as fixed effects, with an interaction term. A random effect was also added for the repeated measures at the animal level. The results were presented as multiplicative factors with their 95% confidence interval.

Preliminary carried out pilot assays were establishing as clinically significant an in-between groups’ difference of use of NE of at least 10 µg/Kg/min. From this standpoint, a main objective of this study was to explore the sparing effect of APLs on NE use during the treatment period following septic shock occurrence, and to a sustained and comparable BP. Consequently, with a level of significance at 5%; and a power of study at 80%, a minimal requested sample size was calculated to at least 6 animals/group using the expected differences of means.

## Results

### Mortality

Mortality was not a measured outcome in this study. Mortality, here defined as the death of a subject prior to the end of the expected 4.5 ± 0.5 h treatment period, was of 33% overall. The surgical procedure itself was associated with a 5% mortality rate, while the Control NE, APL-13 and F13A treatments were associated with 37.5%, 21.4% and 30% mortality rates, respectively. A total of 43 animals contributed to generating the data presented in this section, out of 56 animals initially included in the study.

### Impact of CLP-induced septic shock on cardiovascular function

**Fig. 2:**
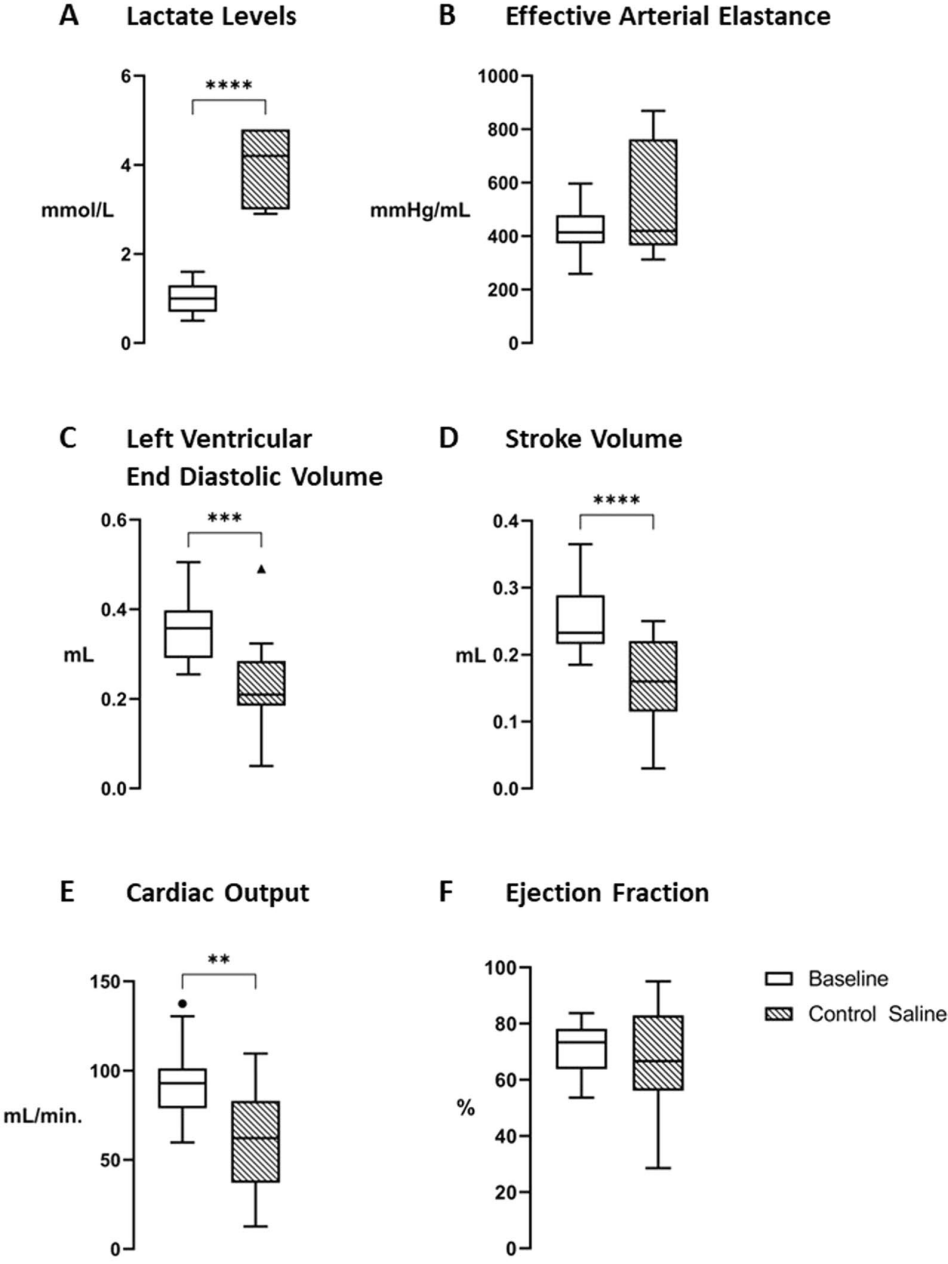
Impact of CLP-induced septic shock on cardiovascular functions. Baseline and septic animals 24 h following the CLP procedure (Control Saline) were compared: **A** Lactate levels (Baseline, *n* = 19, Control Saline, *n* = 7), **B** Effective arterial elastance (Ea) (Baseline, *n* = 18, Control Saline, *n* = 8), **C** Left ventricular end-diastolic volume (LVEDV) (Baseline *n* = 20, Control Saline, *n* = 15), **D** Stroke volume (SV) (Baseline *n* = 20, Control Saline, *n* = 15), **E** Cardiac output (CO) (Baseline *n* = 20, Control Saline, *n* = 15), **F** Ejection fraction (EF) (Baseline *n* = 20, Control Saline, *n* = 15). Data are expressed as mean ± SEM. **A** ****p < 0.0001, using parametric student *t*-tests for paired data. **B-F** *p < 0.05, ** *p* < 0.01, ***p < 0.005, ****p < 0.0001, using parametric student *t*-tests for unpaired data. **A**–**F** “Baseline” = white bars, “Control Saline” = hatched bars

Naïve animals exhibited variations in systolic blood pressure averaging less than 5% of their baseline value. Ninety percent of CLP-challenged rats achieved the requested 20% drop of SBP from baseline. The other ones were excluded, based on the previously mentioned criteria. All baseline values, attributed to the “Baseline” group, were obtained from animals before the CLP procedure. In addition to the anticipated decrease in SBP, CLP-induced sepsis significantly increased lactate levels (*p* < 0.0001 vs. Baseline, Fig. 2A). Left ventricular end-diastolic volume, SV and CO all significantly decreased in septic animals (*p* = 0.0002, *p* < 0.0001 and *p* = 0.0023 vs. [Baseline], respectively. See Fig. 2C–E), indicating impaired cardiac function.

### Effect of NE, APL-13, and F13A co-infusion on hemodynamics in septic rats

**Fig. 3:**
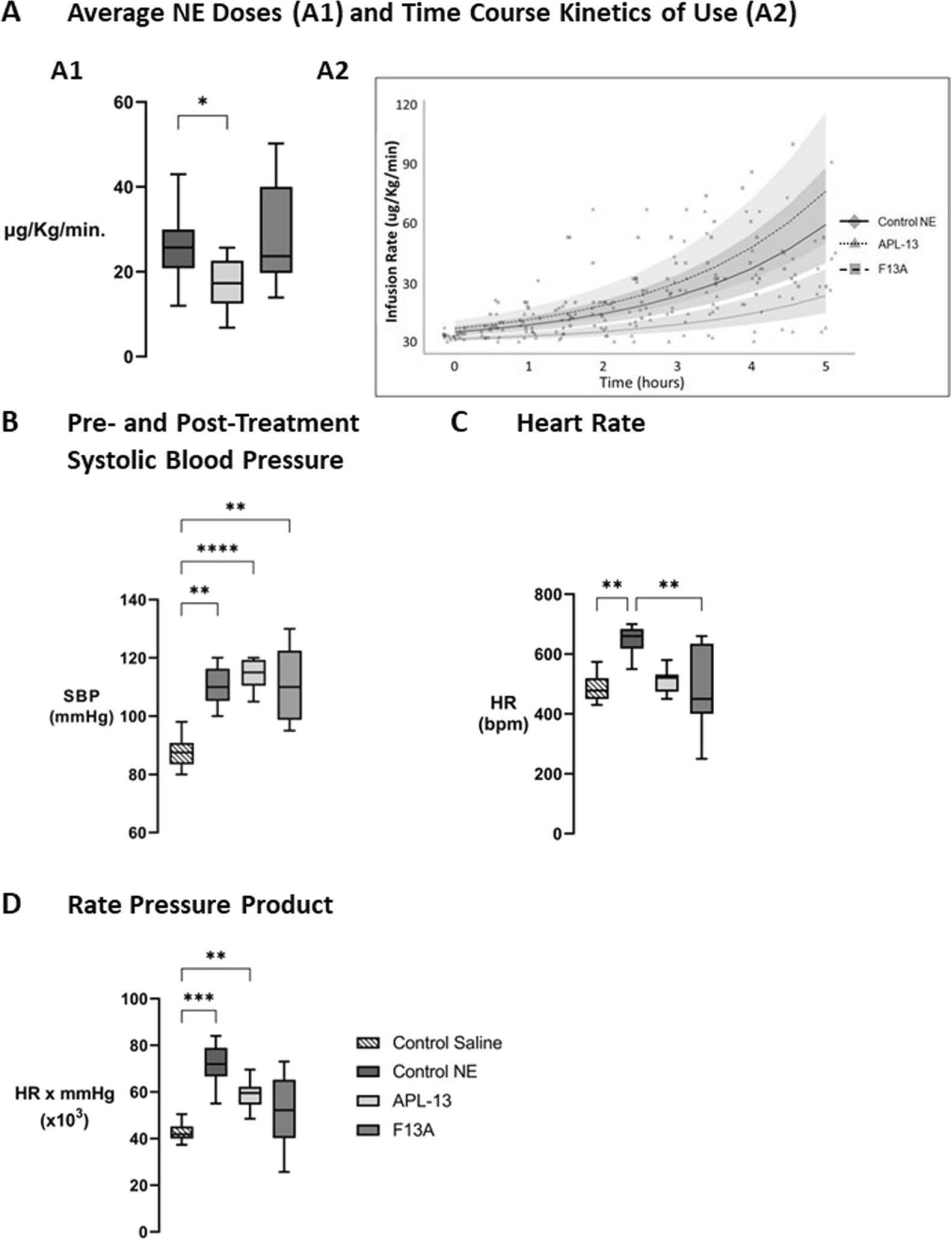
Effect of NE co-infusion with saline; APL-13; or F13A on hemodynamics in CLP septic rats. **A1** Mean NE dose administered to septic animals over a pre-determined treatment period of 4.5 ± 0.5 h. Mean NE dose of CLP rats co-infused with NE + Saline; NE + APL-13; or NE + F13A. **A2** Linear Mixed Model of treatment infusion rates over time, **B** Average post-treatment Systolic Blood Pressure (SBP) obtained with co-infusions. **C** Impact of co-infusions on Heart Rate (HR). **D** Impact of co-infusions on Rate Pressure Product (RPP). Data are expressed as mean ± SEM, *n* = 6–11. **A**–**D**: *p < 0.05, ** *p* < 0.01, ***p < 0.005, ****p < 0.0001 using Dunn’s non-parametric multiple comparisons test. **A**–**D**: “Control NE” = Dark grey boxes (*n* = 10), “APL-13” = Light grey boxes (*n* = 11), “F13A” = Grey boxes (*n* = 7)

There were no differences in treatment durations between the three groups (4.9 ± 0.5 h [Control NE] vs. 5.7 ± 0.7 h [APL-13] vs. 4.79 ± 0.16 h [F13A], *p* = 0.3619). Treatment of septic animals with NE and APL-13 together led to a significant decrease in NE doses required to treat the sepsis-induced decrease in SBP (*p* = 0.0491 vs. [Control NE], Fig. 3A1). Combining F13A to the NE infusion did not impact the cumulative or average dose of NE administered. Linear mixed model analyses indicate that co-infusion of APL-13 with NE is expected to cause a 60% decrease in NE infusion rates over time (*p* = 0.008 vs. NE alone), while co-infusion of F13A is expected to do the opposite, increasing NE infusion rates over time by 218% (*p* = 0.003 vs. APL-13) (Fig. 3A2). As an experimental target, SBP was restored in all groups following the qualifying 20% drop induced by CLP, with no difference in post-treatment SBP (Fig. 3B). Animals treated with NE alone displayed a higher HR compared to animals treated with NE and F13A (*p* = 0.0092, Fig. 3C). Animals treated with NE and APL-13 displayed almost no change in HR compared to pre-treatment values. Animals treated with NE alone and in concomitance with APL-13 showed significant increases in RPP (*p* = 0.0001 and *p* = 0.0067 vs. [Control Saline], respectively, Fig. 3D).

### Cardiovascular benefits of APL-13 as an adjuvant drug to NE in septic shock

**Fig. 4:**
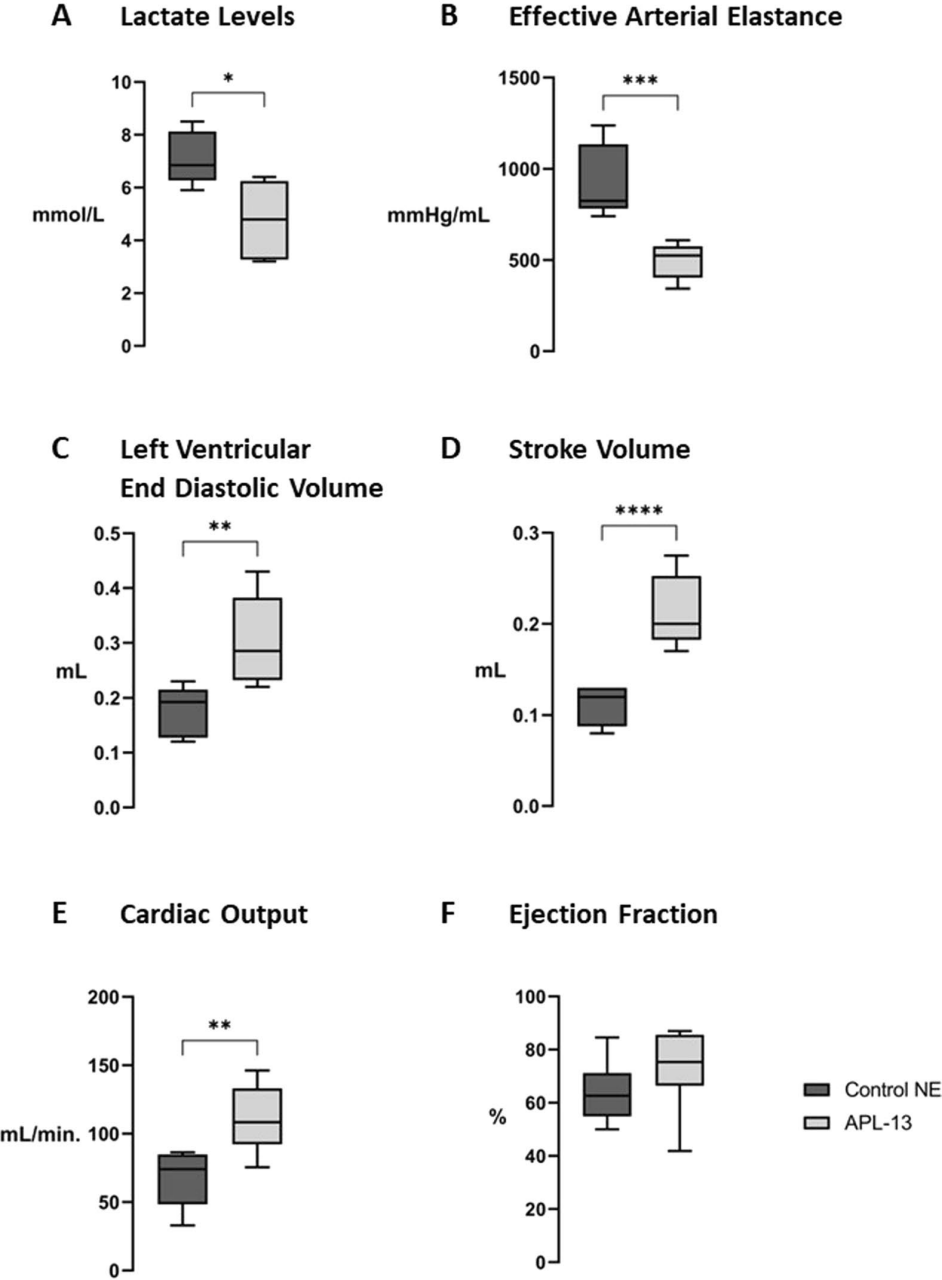
Effect of NE co-infusion with saline vs. APL-13 on lactate levels and cardiac parameters in CLP septic rats. Impact of NE Administration, in concomitance with NS or APL-13, on **A** Lactate levels, **B** effective arterial elastance (Ea), **C** left ventricular end-diastolic volume (LVEDV), **D** stroke volume (SV), **E** cardiac output (CO) and **F** ejection fraction. Data are presented as mean ± SEM, *n* = 6 to 8. **A–C**: *p < 0.05, **p < 0.01, ***p < 0.005, ****p < 0.0001, using non-parametric student’s *t* tests (Mann Whitney) for unpaired data. **A**–**C**: “Control NE” = black bars, “APL-13” = dark grey bars

Co-infusion of APL-13 with NE significantly lowered (i) blood lactate levels (*p* = 0.0108 vs [Control NE], Fig. 4A) and (ii) Ea (*p* = 0.0007 vs [Control NE], Fig. 4B), while overall cardiac functions were improved by APL-13 administration: following the treatment period, septic animals treated with NE and APL-13 displayed significantly improved LVEDV (*p* = 0.0020 vs [Control NE], Fig. 4C), SV (*p* = 0.0007 vs. [Control NE], Fig. 2D) and CO (*p* = 0.0040 vs. [Control NE], Fig. 2E).

## Discussion

Concepts of decatecholaminisation and catecholamine-sparing strategies, as well as the need to personalize treatment during septic shock treatment, are a current debate amongst experts [25–28]. These are based on evidence suggesting an increased frequency of adverse cardiac events, more organ dysfunction, and higher mortality risks in patients with higher catecholamine loads, as well as the well-documented cardiotoxic effects of this family of drugs [16, 29, 30]. Vasopressin has been one of the pioneer drugs in this approach, allowing mild benefits and no improvement mortality-wise [31, 32]. Angiotensin II was also recently approved by the FDA as a treatment for hypotensive shock, although it should only be used in patients not responding to high doses of catecholamine or vasopressin [33]. Alternatively, the use of β-blockers and vasodilators (especially those associated with improvements in cardiac function) during septic shock management were suggested to be cardioprotective and partly reverse SIMD symptoms [34, 35]. However, the impact of such β-blockers on mortality risks in septic shock patients is still debated [36, 37]. The apelinergic system was shown to improve cardiovascular function in healthy animals and in septic conditions [23]. We demonstrated in this study the potential of APL-13 in reducing NE doses to maintain appropriate BP, while improving cardiac function through an improved ventriculo-arterial coupling [38] and with a reduced workload for the heart in septic animals.

### NE-sparing effect of APL-13 in septic rats

Administration of APL-13 decreased the amplitude of NE dose increments required to maintain appropriate SBP in septic rats, leading to statistically significant reductions in cumulative NE doses received by septic rats over a treatment period of 4.5 ± 0.5 h. All intended to treat groups presented similar post-treatment SBP values despite a 43% difference in cumulative NE doses. On the contrary to septic rats infused with APL-13, those treated with the APJ antagonist F13A did not lead to significantly lower cumulative NE doses than those treated with NE alone. Whether this is related to a partial agonist/antagonist activity of F13A; to a decoupling impact of blocking endogenous APLs on catecholamine’s drive and needs; is beyond the scope of this study.

The NE-sparing effect of APL-13 translates into a potentially improved prognosis, in the form of significantly lower blood lactate levels. Catecholamine doses have been shown to positively correlate with blood lactate levels, disease severity and mortality during septic and cardiogenic shock, and have a better prognostic accuracy than that of the qSOFA score [39–41]. The decreased sympathetic activity associated with lower NE doses, as well as the reduced cardiac workload associated with APL-13 treatments, are potential causes for the decreased lactate levels observed in animals treated with NE and APL-13 in our study.

### Cardiovascular effects of NE-APL-13 co-infusion

Decreased NE doses, in combination to APL-13 administration, were associated with improved cardiac diastolic and systolic functions. High HR values are linked to increased energy demands and oxygen consumption, but also with lower diastolic period durations, impacting ventricular filling [42]. The NE-sparing effect of APL-13 was associated with lower HR, leading to improved ventricular filling and potentially improved coronary perfusion, while reducing cardiac stress and oxygen consumption (as indicated by RPP and Ea) [43, 44]. The observed improvement in LVEDV may be the result of both decreased HR and preserved plasma volume [22, 23].

The HR displayed by animals treated with F13A is unexpected, given the cumulative NE dose those animals received, and the difficulty level of acquiring echocardiographic data in these animals (high sensitivity to anesthesia and high mortality during the procedure, see study limitations). Given our group’s history with F13A and its impact on cardiac function, the current data may need more investigation in future studies [23]^.^

The impact of APL-13 on diastolic function, along with the improvements in systolic function observed in animals treated with APL-13 and NE, go along with the positive inotropic effects of APL-13 previously reported by our group and others [22, 45].

## Study limitations

A CLP model in rats is not a clinical septic shock in humans. The relative low fluid resuscitation protocol selected in this experimental model was lower than in other CLP-induced septic shock studies [46, 47] but can be justified by recent findings suggesting a more restrictive fluid resuscitation did not result in significantly higher morbidity/mortality [11]. The attribution of the infusion treatment at the inclusion period was not blinded. The duration of the treatment period is arguably short, a limitation associated in part to the ethical obligations associated with the project. On the other hand, it is the Surviving Sepsis Campaign’s (SSC) recommendation that vasopressors be administered early to rapidly improve blood pressure, and while experts are still debating the benefits of alternative vasopressors, studies suggest that vasopressin, recommended as an alternative vasopressor by the SSC, presents more benefits in less severe cases of septic shock. The early phases of treatment thus seem like the appropriate window of time to introduce alternative therapies, but more importantly, benefit from them [48, 49]. Monitoring of fluid intake and urine output would have provided the necessary data to assess of the appropriateness of the fluid resuscitation approach applied in this study, as well as an indication of intravascular volume preservation. The impact of sex on cardiovascular response was neither evaluated nor compared in this experimental design. Quantification of organ and tissue injury biomarkers would have been relevant given the importance of organ damage in the pathophysiology of septic shock, but the protective effect of APL-13 when compared to traditional catecholamine therapy has already been demonstrated [11]. Because of a rapid decline in the animals’ state following anesthetic induction, precluding any measurements, echocardiographic data could not be thoroughly and regularly secured in the F13A co-infused group. Although originally described as an inhibitor counteracting the cardiovascular benefits associated with APL-13 [50], F13A could have additionally impaired cardiac functions, as already observed in a “septic-like” endotoxic model [23]. As to other vital organ damages, F13A was reported protective and regenerator for liver and kidney in non-septic conditions [51–54]. As it stands and at this time, it remains impossible to formulate a conclusive statement on the role of F13A in sepsis-related cardiovascular dysfunction and organ damage, beyond the available evidence. Furthermore, one should note that the intra-cardiac pressure was not measured due to the invasive nature of the procedure, but rather estimated from the systemic arterial pressure. Values of Ea displayed should be considered as estimated Ea. The choice of agonist and dose was made based on prior experiments by our group but incorporating more doses of APL-13 would have allowed us to verify the existence of a dose–response relationship when it comes to the NE-sparing effects we observed. Also, the inclusion of other apelin receptor -APJ- agonists (i.e., APL-17, APL-36, Ela) would have helped in identifying the best agonist, amongst the currently studied ones, when it comes to cardiovascular improvements and NE-sparing in the context of septic shock.

## Conclusion

In the presented experimental conditions, introduction of APL-13 as an adjuvant molecule for the treatment of sepsis-induced hypotension allowed for important reductions in cumulative NE doses administered, while efficiently supporting hemodynamics. APL-13 led to improved cardiovascular functions and workload “environment”, likely responsible for the reduced requirements in NE doses. Moreover, APL-13 reduced parameters of mortality risks, such as lactate levels.

## Data Availability

The datasets used and analyzed in this current study are available from the corresponding author on reasonable request.

## Abbreviations

APJ: Apelin receptor
APLs: Apelins
APL-13: Apelin-13
BP: Blood pressure
CO: Cardiac output
CLP: Cecal ligation and puncture
DOB: Dobutamine
DBP: Diastolic blood pressure
Ea: Arterial elastance
Elabela: Ela
ESP: (Left ventricular) end-systolic blood pressure
F13A: Apelin F13A, [Ala]-Apelin-13
HR: Heart rate
LVdP: Left ventricular developed pressure
LVEDV: Left ventricular end diastolic volume
LVESV: Left ventricular end systolic volume
NE: Norepinephrine
NS: Normal saline
PP: Pulse pressure
RPP: Rate pressure product
qSOFA: Quick sequential organ failure assessment
SBP: Systolic blood pressure
SIMD: Sepsis-induced myocardial dysfunction
SV: Stroke volume
